# Supplementary material for: Robust and highly efficient transformation method for a minimal mycoplasma cell
Source: J Bacteriol. 2025 Feb 4;207(3):e00415-24. doi: 10.1128/jb.00415-24 (PMC11925241; doi:10.1128/jb.00415-24)
Supplement: Supplemental figures — Fig. S1 to S4. [file jb.00415-24-s0001.pdf]

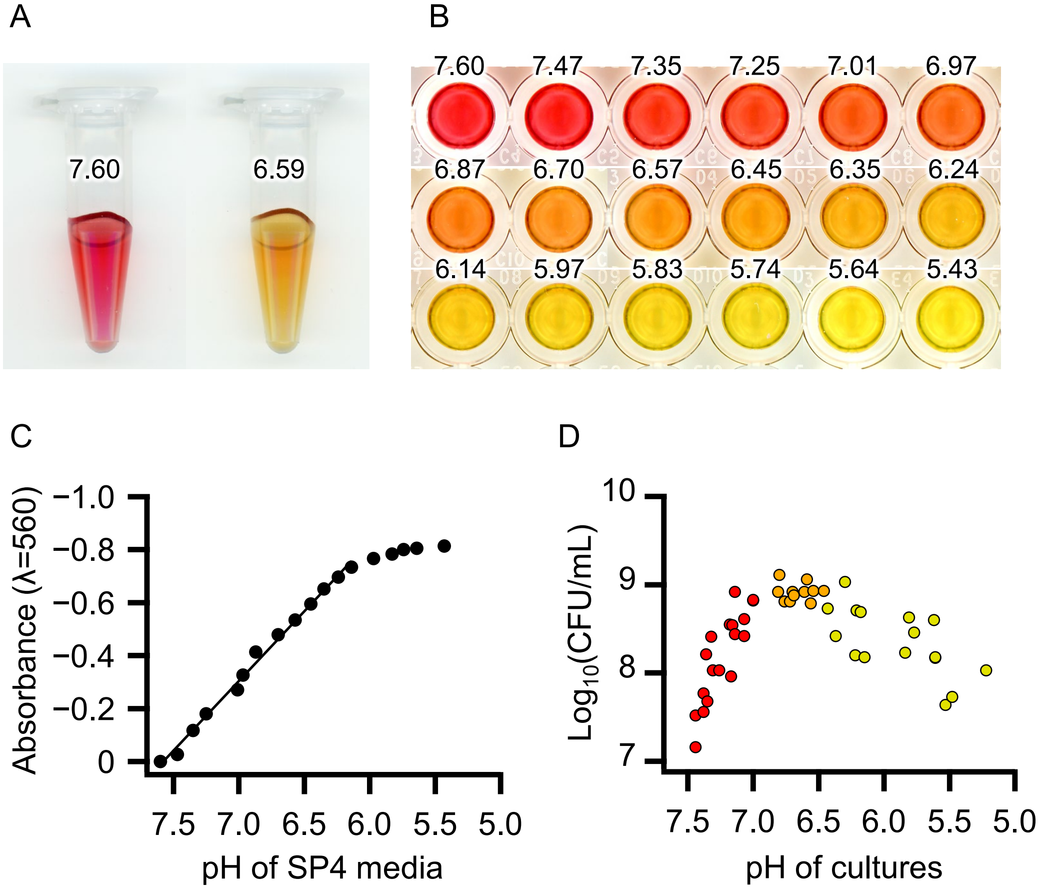

SUPPLEMENTARY FIGURE S1.

pH and color change of SP4 media and number of JCVI-syn3B cells during cultivation.

(A) Color changes during cultivation of JCVI-syn3B in a 1.5 mL tube. The left and right tubes are before and after cultivation. The pH of each culture is also shown. (B) Color change of SP4 medium under various pH conditions. The series of SP4 media were prepared by addition of HCl. The pH of each medium is shown at upper of each well. (C) Light absorbance at 560 nm in a series of SP4 media at various pH. The pH and absorbance were linearly correlated within the range of pH 7.6–6.1 ($R^2 = 0.99$). (D) The number of viable cells (CFU) at various pH of cultures. The growth phase after the lag phase could be divided into three phases: I, II, and III, represented by red, orange, and yellow, respectively. These colors are the same as those in Figure 3.

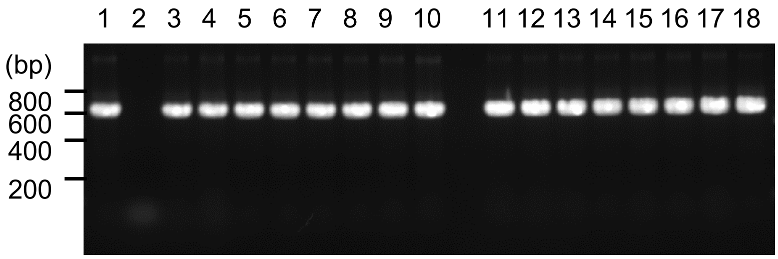

SUPPLEMENTARY FIGURE S2.
Colony PCRs to confirm transformation of JCVI-syn3B cells.
The puromycin resistance gene was amplified. Lane 1: positive control (JCVI-syn3B strain containing the puromycin resistance gene). Lane 2: negative control (wild type JCVI-syn3B strain). Lanes 3–10 and 11–18 were transformants with pSD128 and pSD131, respectively.

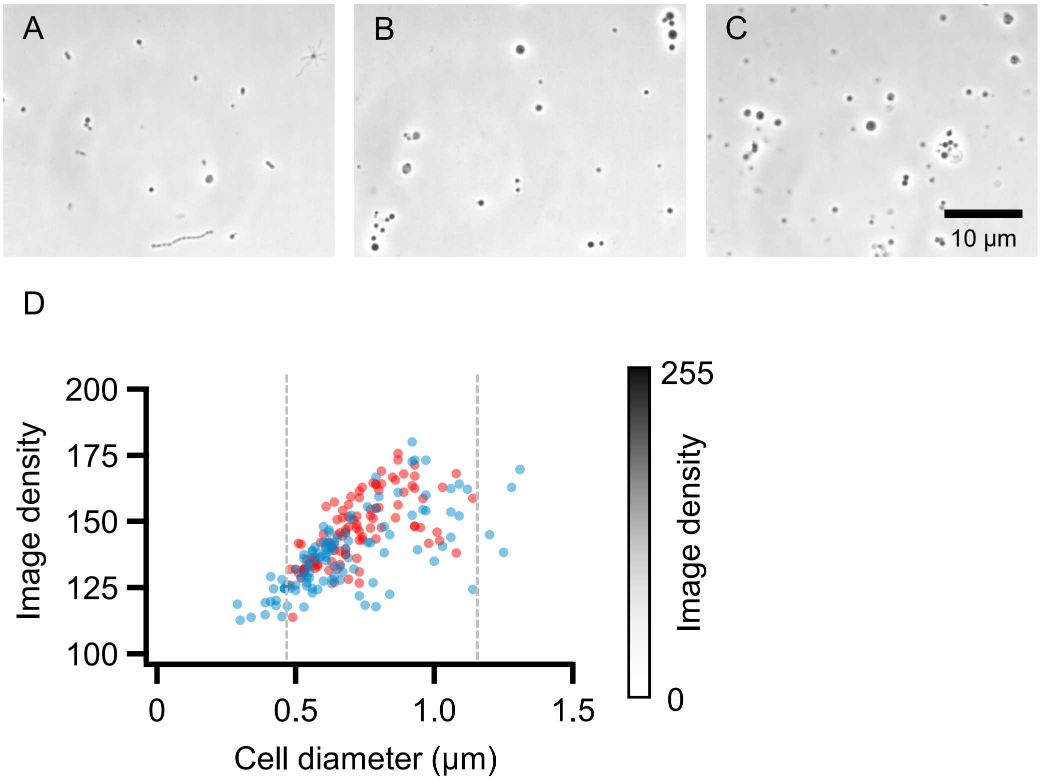

SUPPLEMENTARY FIGURE S3.
Morphology of JCVI-syn3B competent cells.
Phase-contrast micrographs of (A) intact JCVI-syn3B cells, (B) non-frozen competent JCVI-syn3B cells, and (C) frozen competent JCVI-syn3B cells that were kept at −80° C for 7 days and then thawed. (D) Diameter and image density of individual cells were measured and plotted. A total of 105 and 111 cells from non-frozen and frozen competent cells were measured and plotted in red and blue, respectively. The legend for image density is shown on the right. Dashed gray lines indicate the maximum and minimum diameter of non-frozen competent cells. The mean diameter of the cells, mean image density, and their significance test (*t*-test) are shown in Supplementary Table S7.

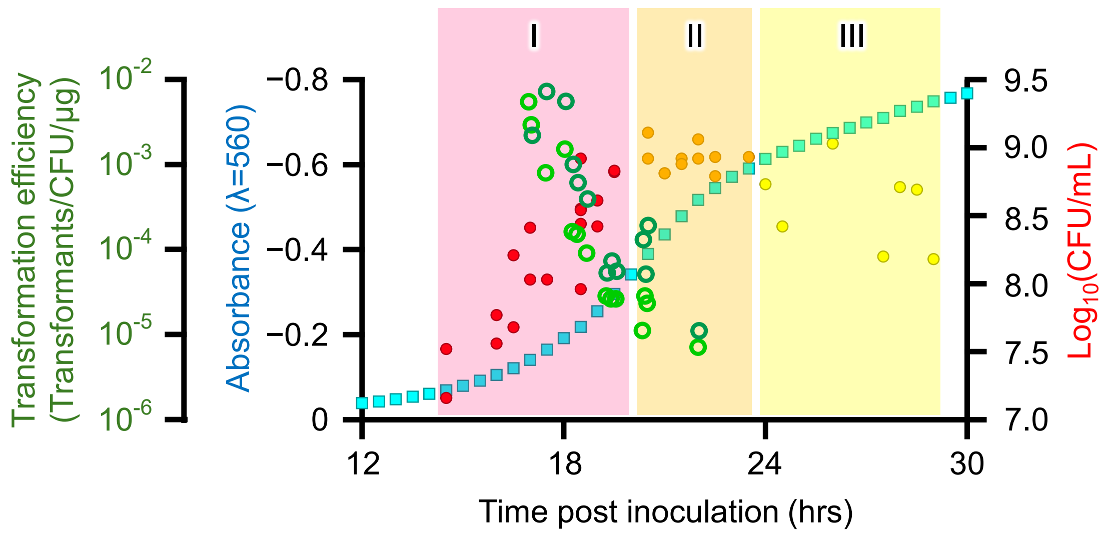

SUPPLEMENTARY FIGURE S4.

Summary of growth rate, number of cells (CFU), and transformation efficiency of JCVI-syn3B.

Three kinds of data are overlaid: the growth rate (absorbance at 560 nm, squares in light blue) and CFU (filled circles) of JCVI-syn3B cells are the same as in Figure 3, and the transformation efficiencies are the same as in Figure 4. Only the data around the exponential phase are displayed. The transformation efficiencies with 100 ng of pSD128 and pSD131 plasmids are shown in open circles in light green and dark green, respectively. There are three different *X*-axis scales, each labeled with colors similar to the data plots. High transformation efficiency was observed in the early-exponential phase.
